# Preliminary validity of a daily functional status pain assessment tool

**DOI:** 10.1093/jscdis/yoaf006

**Published:** 2025-02-19

**Authors:** Wally R Smith, Rehan Qayyum, Alexandra Ulbing, Margaret S Guy, Daniel M Sop, Yue May Zhang

**Affiliations:** Division of General Internal Medicine, Department of Internal Medicine, Virginia Commonwealth University, Richmond, VA 23219-0306, United States; Department of Internal Medicine, Macon & Joan Brock Virginia Health Sciences at Old Dominion University, Norfolk, VA 23501-1980, United States; Department of Physical Medicine and Rehabilitation, School of Medicine, Virginia Commonwealth University, Richmond, VA 23298-0677, United States; Division of Hospital Medicine, Department of Internal Medicine, Virginia Commonwealth University, Richmond, VA 23298-0102, United States; Sickle Cell Disease Program/Division of General Internal Medicine, Department of Internal Medicine, Virginia Commonwealth University, Richmond, VA 23298, United States; Department of Biomedical Engineering, Virginia Commonwealth University, Richmond, VA 23219-0306, United States; Sickle Cell Disease Program/Division of General Internal Medicine, Department of Internal Medicine, Virginia Commonwealth University, Richmond, VA 23298, United States

**Keywords:** SCD, pain, functional assessment, quality of life

## Abstract

**Objectives:**

Readiness for discharge for a SCD vaso-occlusive crisis is dictated by factors far beyond pain control, including physical function/activity. We therefore designed and tested a functional status-based pain assessment questionnaire in SCD patients hospitalized with vaso-occlusive crises.

**Methods:**

Sickle cell disease patients on a preselected nursing unit rated 10 draft Functional status-Based Pain Assessment items of activities of daily living on a five-point Likert scale (0-5) from “very easy” to “very difficult” daily on each day of their admission until discharge, at approximately the same time. Concurrently, they reported Numeric Rating Scale (0-10) pain intensity. For validation, we used exploratory factor analysis, confirmatory factor analysis, and item response theory analysis.

**Results and discussion:**

We analyzed 503 observations from 175 admissions of 88 patients. Half were female, the mean age was 32.1 ± 11.8 years, and the mean length of stay was 7.1 ± 6.9 days. The mean Numeric Rating Scale (6.8 ± 1.9) was inversely correlated with the mean Functional Status-based Pain Assessment (0-50) score (27 ± 8.0, *r* = −0.4342, *P* < .0001). Functional Status-based Pain Assessment item means ranged from 2.1 to 3.3. Cronbach’s alpha was 0.91. Exploratory factor analysis showed that all Functional Status-based Pain Assessment items loaded on a single factor. Confirmatory factor analysis found adequate convergent and discriminant validity and showed strong fit of the model to the data. Item response theory analysis showed item discrimination ranging from 0.56 to 4.1, while difficulty ranged from −2.8 to 7.5.

**Conclusion:**

The Functional Status-based Pain Assessment shows strong correlation with daily Numeric Rating Scale, is multidimensional, and demonstrates strong construct validity. It may improve assessment of SCD vaso-occlusive crisis pain and may enhance vaso-occlusive crisis discharge discussions.

## INTRODUCTION

Sickle cell disease, the most common set of genetic hemoglobinopathies worldwide, primarily affects patients of African and Mediterranean descent,^1^ including approximately 100 000 Americans.^2^ It is caused by single-point mutation leading to the substitution of valine for glutamic acid in the sixth position of the beta globin chain, *HBB*.^3^ It is associated with unpredictable pain due to severe vaso-occlusion, often requiring opioids and sometimes provoking hospitalizations. In western countries, the frequency of vaso-occlusive crises (VOCs) provoking hospitalization is a predictor of morbidity and mortality.^4^

Given the outsized impact of SCD as a cause for hospital admissions in the United States,^5^ clinicians and hospital administrators struggle to avoid SCD admissions and to get admitted SCD patients discharged as soon as medically appropriate. Frequent pain reassessment and early titration of opioid and other analgesics, especially within the first 24 hours, is the cornerstone of lowering hospital length of stay for inpatients with SCD.^6^ But these pain assessments, which focused on appropriateness of hospitalization or discharge, are fraught with measurement issues.

Evaluating when hospitalization for patients with SCD is warranted and when discharge from the hospital is appropriate is fraught with measurement issues. In-hospital SCD pain is commonly clinically evaluated serially using a unidimensional pain intensity rating. Most commonly, evaluators use the Numeric Rating Scale (NRS) using an 11-point scale (0-10), and asking patients to choose a single number to represent pain intensity either at that moment, at their worst during the past 24 hours, or their average pain during the past 24 hours.

However, pain is a multidimensional experience consisting of intensity, frequency, location, character, and multifaceted pain responses. The unidimensional NRS pain intensity rating is often swamped by or ignores these characteristics. Further, evaluation of pain is often swamped by environmental variables, including patient psychosocial and economic variables, hospital access, and pain response variables. This makes pain management decisions and evaluation of readiness for discharge during a hospitalization for a SCD VOC difficult. Daily judgments and daily negotiations between patients and their inpatient caregivers usually determine readiness for discharge from the hospital for a VOC.

In addition, the choice of pain discharge readiness assessment might strongly affect endpoints in clinical trials. For example, a recent phase III trial of Rivipansel, a promising selectin inhibitor for remission of pain in SCD VOC, which had demonstrated positive phase II results,^7^ failed to show a difference in median time to readiness for discharge, the primary end point. Similarly, no differences were found in time to actual discharge, time to discontinuation of intravenous opioids, and cumulative intravenous opioid use.^8^

Evaluation of parameters other than pain should augment clinician decision-making in this context. But respondent burden imposed by this evaluation could be high for uncomfortable pain patients. They may refuse, or at least not like being asked while suffering to complete lengthy inpatient multidimensional pain and biopsychosocial scales that incorporate concepts like physical function, stress, social support, or environmental influences.

We therefore sought to determine whether a formal, brief inpatient assessment that included physical function dimensions was feasible in inpatients with SCD. We also sought to determine whether responses would be valid. While we felt the survey would be useful to anyone in pain with an acute-on-chronic painful disease, we focused this survey on evaluating patients with SCD. We knew of an existing pediatric daily functional pain SCD scale and that it had not been validated among adults. We also reasoned that adults would have dimensions of function different than those of youth, so they would need a different scale.

Therefore, we developed a survey, then drafted and tested items meant to compose the Functional Status-Based Pain Assessment (FSPA) scale in adult SCD patients hospitalized with VOC. One goal of scale development and administration was to demonstrate feasibility of completing the survey without overburdening patients with questions. A second goal was to show the internal validity of the scale for measuring daily function. An eventual goal was to enhance the daily patient/physician dialog about readiness for discharge during VOC.

## MATERIALS AND METHODS

### Sample

The study was conducted on 1 preselected nursing unit from January 2018 to June of 2019. Patients who were nonverbal or functionally not able to complete form were excluded. The collected sample, prior to exclusions, was of adequate size for validation of an 11-item quantitative survey, based on published guidelines.^9^

### Patient safety, institutional review

Assessments were used immediately to guide daily patient care. We submitted this study to the VCU IRB, and it was ruled as an exempt survey study for quality improvement in 2018. Surveys were administered to all SCD patients admitted on a preselected nursing unit, which preferentially selected SCD admissions from January 2018 to June of 2019.

### Procedures measures

Procedures consisted of nursing staff providing paper surveys to patients for self-assessment, then collecting them for analysis and interpretation by the team. Patients were asked to complete procedures daily at approximately the same time of day. Subsequently, a chart review was performed by one of us (D.M.S.) to attempt to phenotype patients. The chart review encountered challenges in retrieving data, especially laboratory data, and matching it with patient ID, since this was a quality improvement study. The difficulty was magnified by a switch from 1 electronic health record vendor to a second during the stay. Admissions that occurred before the new vendor system was installed proved difficult to access for laboratory records, due to incomplete mapping of legacy files from the legacy electronic health record. This often left a mismatch. The most recent admission’s laboratory data, extracted from the new EHR, did not always match the NRS and FSPA data, collected during a previous admission using the legacy EHR.

### FSPA items and summary score

**Figure 1.**
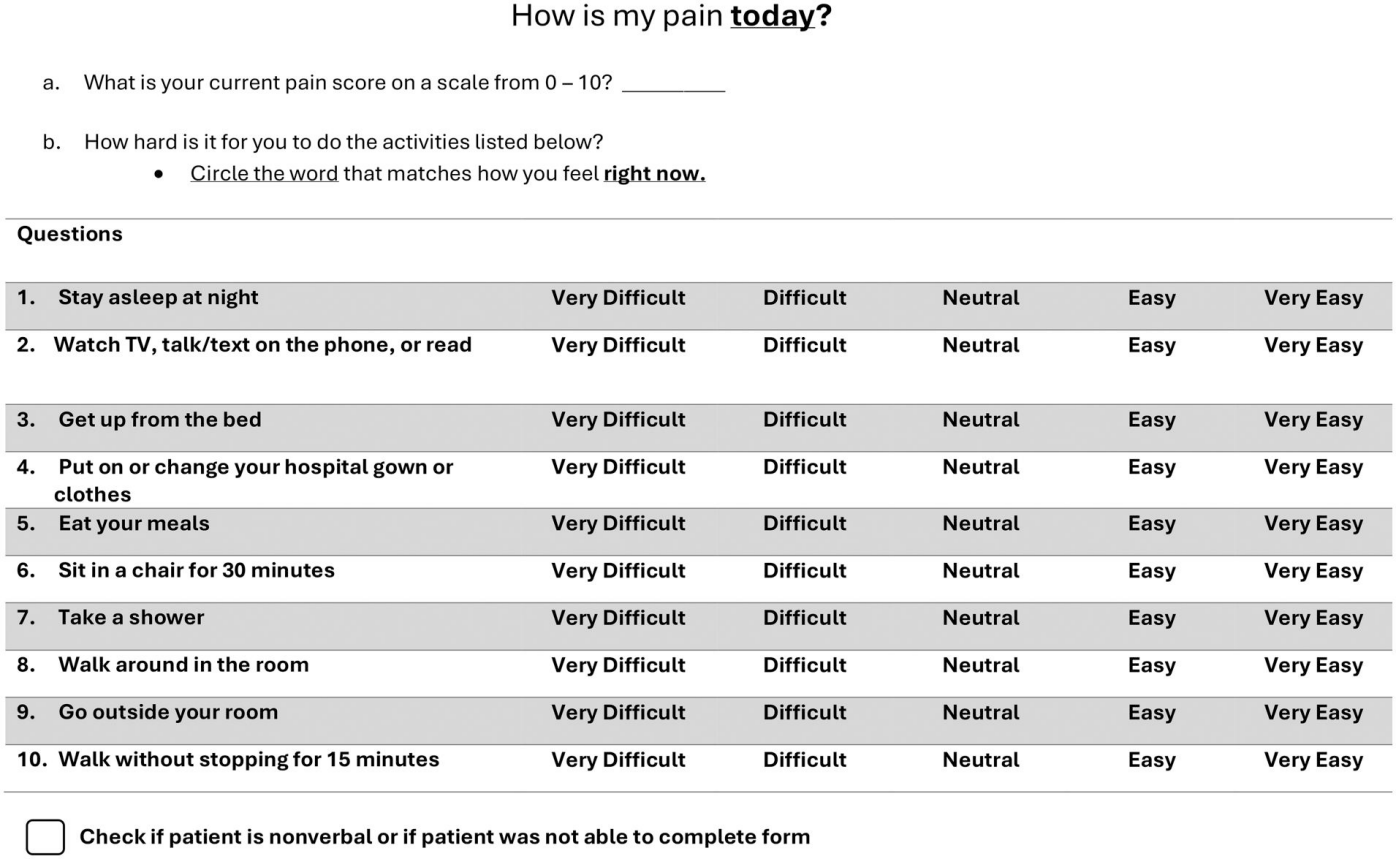
Final Functional Status-based Pain Assessment questionnaire.

FSPA items were drafted using concepts from existing scales. While a few items were relied heavily on Zempsky et al.,^10^ who developed the only inpatient functional pain assessment tool for SCD we found, the Youth Acute Pain Functional Ability Questionnaire (YAPFAQ), plus input from inpatient management experts familiar with SCD, including physicians, nurse practitioners, pharmacists, and bedside nurses. Items comprised tasks that were common during hospital stays and tasks considered essential to allow hospital discharge. Activities included sleeping, watching TV, walking around the room, or eating a meal in a chair. Each item required the patient to self-report ability on a five-point Likert scale ranging from “very easy” (5) to “very difficult” (1). The survey was designed at a health literacy grade of 1 (Figure 1).

Each item of the FSPA required an ordinal response, graded on an ordered scale of 1-5. We drafted 10 items. Thus, the range of possible FSPA scores was from 10 to 50. The assessment was evaluated and found to be on a health literacy grade of 1 and was translated into Spanish, Arabic, and Chinese.

### Numeric rating scale

Concurrent to the survey, patients also provided pain scores on a NRS (0-10).

The NRS for pain intensity has been described above. An 11-item NRS scale (0-10) was used.

### Analysis

#### Item response theory analysis

We used item response theory (IRT), a psychometric approach commonly employed in educational testing, to assess the comprehensiveness of the FSPA in capturing the entire range of pain-related functional impairment in sickle cell patients. In the context of our study, IRT was applied to estimate the latent ability (pain control) of sickle cell patients based on their responses to items representing varying levels of difficulty related to functional capacity. We reasoned that, analogous to students, respondents (in this case, patients with SCD) exhibited differing abilities (pain expression and pain function), determined by their responses to survey items. We viewed SCD patients’ differing function and pain expression would be reflected by their lower likelihood of achieving increasingly more difficult-to-achieve tasks. We predicted patients would accurately and consistently respond to items rating this functionality, from easy to difficult.

We built a rating scale model, a partial credit model, a generalized partial credit model, and a graded response model (GRM), and selected a GRM model, based on the Akaike information criterion and Bayesian information Criterion. GRM is an extension of the two-parameter IRT model designed for ordered responses and ascertained the difficulty and discrimination parameters for each item in the FSPA questionnaire. The difficulty parameter denotes the position of an item on the pain control scale where half of the respondents can answer it. Discrimination, on the other hand, measures the rate at which the probability of answering an item changes in relation to pain control near the item’s difficulty; an item with a steeper slope exhibits a higher discrimination parameter and can more effectively differentiate between low and high levels of pain control. The GRM characterizes each item with its unique discrimination parameter and features cut points that delineate the thresholds between the ordered outcomes. These cut points represent the difficulty of responding with a specific category or higher for a given item. Consequently, the GRM model is analogous to an ordered logistic model.

Discrimination for each item was judged using prior publications as: none (0); very low (0.01-0.34); low (0.35-0.64); moderate (0.65-1.34); high (1.35-1.69); very high (≥1.7), or; perfect (+ infinity).^11^ Lastly, we calculated the model-predicted expected score for the whole instrument for each patient and graphed it against the NRS score to obtain a test characteristic curve.

#### Comparisons, modeling of FSPA versus mean pain on NRS

Further, we modeled the fit of the FSPA score to mean pain on the NRS by calculating means (SDs) or frequencies of all patients’ daily pain intensity NRS scores as well as the mean of their FSPA scores. We then used Pearson’s correlation to examine the relationship between these 2 continuous variables.

#### Cronbachs’ alpha

Reliability of the FSPA scale was measured by a conventional standard, the Cronbach’s alpha. The coefficient alpha is the reliability coefficient, a measure of the internal consistency of tests and measures.^12^

#### Exploratory and confirmatory factor analysis

We used Exploratory factor analysis (EFA) using a principal component factor analysis (PCFA) without rotation to explore the underlying structure of the data. Pairwise deletion was used for any missing values. We used Eigenvalues and visual examination of scree plot to assess the number of retained factors. We did perform PCFA, but results were consistent with our conceptual hypothesis that all the items of the FSPA tap into 1 dimension, which we labelled “functional status contributory to readiness for discharge.”

The initially identified factor structure through EFA was tested using confirmatory factor analysis (CFA), with listwise exclusion of missing data. Factor loadings were examined, and the overall model fit was assessed by evaluating the model χ^2^, comparative fit index (CFI), root-mean-square-error of approximation (RMSEA), and standardized root-mean-squared residual (SRMR). Optimal fit criteria for these indices include non-significant χ^2^ tests, CFI exceeding 0.95, SRMR values <0.05, and RMSEA values <0.05. Of note, the model χ^2^ tests are highly sensitive to minor deviations from an ideal fit and sample size, often yielding statistically significant results even for well-fitting models. To address potential areas of misfit, improvements were made by introducing covariances between select residuals in the measurement structure.

All analyses were performed using Stata 14.1 (College Station, Texas).

## RESULTS

Table 1 lists patient demographic characteristics for the sample. The original quality improvement dataset consisted of daily observations from 217 unique admissions. However, 25 of these admissions were not associated with an admission identifier, and an additional 12 were not associated with an identifiable patient. This left 135 unique patients. We excluded patients with missing NRS pain intensity scores during days 1 and/or 2 of their hospital stay. This left 503 daily observations from 175 unique admissions of 88 unique patients as the analysis dataset. Because the surveys were administered on a clinical ward by clinicians (see Acknowledgements), we failed to gather a strict denominator of approaches and acceptances. Further, we did not survey patients about the respondent burden of the survey or their attitudes toward the survey. However, anecdotally, we had a few refusals among the approaches made.

**Table 1.** Demographics and baseline clinical characteristics of sample, *N* = 88

Age (years), mean and SD^a^	32.1 ± 11.8
Race, Black %	100
Gender %	
Male	36
Female	45
Unknown, not reported	7
Average length of stay (says ± SD)	7.1 **±** 6.9
Genotype %	
HbS Beta +	6
HbS beta 0	5
HbSC	19
HbSG Philadelphia	1
HbSS	43
UNK	14

a Data missing for 7 participants.

Table 2 lists the candidate functional domains and items, which, to obtain parsimony, were voted on by SCD experts (including some coauthors and those listed in the Acknowledgments), who met regularly to discuss appropriate coverage for the questionnaire. Figure 1 shows the final FSPA questionnaire.

**Table 2.** Functional pain domain and item candidates.

Domain	Source of candidate domain
Wash your body?	Zempsky 2014, item 15
Wash or shampoo your hair	Zempsky 2014, item 18
Put on or change pants	Zempsky 2014, item 26
Take a shower?	Zempsky 2014, item 1
Put on or change your hospital gown or clothes?	Zempsky 2014, item 6
Get up from the bed?	Zempsky 2014, item 16
Walk around in the room?	Zempsky 2014, item 17
Go outside your room?	Zempsky 2014, item 19
Be up without needing to rest?	Zempsky 2014, item 20
Turn over or roll over in bed?	Zempsky 2014, item 36
Get to sleep at night?	Zempsky 2014, item 10 (reworded)
Stay asleep at night?	SCD Expert opinion
Climb 1 flight of stairs?	Ware MOS-SF-36, item 7
Sit in a chair for 30 minutes?	Zempsky item 21 (adapted)
Walk continuously for 15 minutes?	Expert Opinion
Watch TV, talk/text on phone, or read?	Zempsky 2014, items 3, 23,
Talk to family or friends?	Zempsky 2014, item 34
Eat your meals?	SCD Expert opinion

Table 3 shows the FSPA Item means, the NRS pain score means for all observations, and correlations between each of these items. The FSPA mean was 27 ± 8.0. The mean NRS score for all responses (range 0-10) was 6.8 ± 1.9. The FSPA item means ranged from a low of 2.1 (item “Walk without stopping for 15 minutes”) to a high of 3.3 (item “Watch TV, talk/text on the phone, or read”). Correlation between many item pairs was high, except for correlation with sleep, suggesting that the 2 items tapped a similar concept, or they were strongly related.

**Table 3.** FSPA parameters and means, NRS mean, correlations.

	Mean (SD)	Pain score	Sleep	TV_Phone	Out of bed	Hops-Gown	Meals	Sit	Shower	Walk	Outside	Walk_15
Pain score	6.80 (1.92)	1										
Sleep	2.16 (0.96)	−0.37	1									
TV_Phone	3.28 (1.01)	−0.26	0.39	1								
Out of bed	2.69 (1.05)	−0.32	0.29	0.46	1							
Hops_Gown	2.77 (1.05)	−0.37	0.23	0.45	0.72	1						
Meals	3.17 (1.01)	−0.25	0.25	0.46	0.52	0.48	1					
Sit	2.84 (1.12)	−0.35	0.23	0.42	0.57	0.60	0.50	1				
Shower	2.60 (1.08)	−0.33	0.27	0.41	0.57	0.65	0.40	0.56	1			
Walk	2.72 (1.15)	−0.32	0.19	0.40	0.64	0.65	0.40	0.60	0.66	1		
Outside	2.59 (1.15)	−0.35	0.21	0.39	0.66	0.65	0.47	0.60	0.69	0.76	1	
Walk_15	2.14 (1.15)	−0.33	0.23	0.31	0.59	0.58	0.38	0.52	0.63	0.65	0.73	1

### IRT analyses

**Figure 2.**
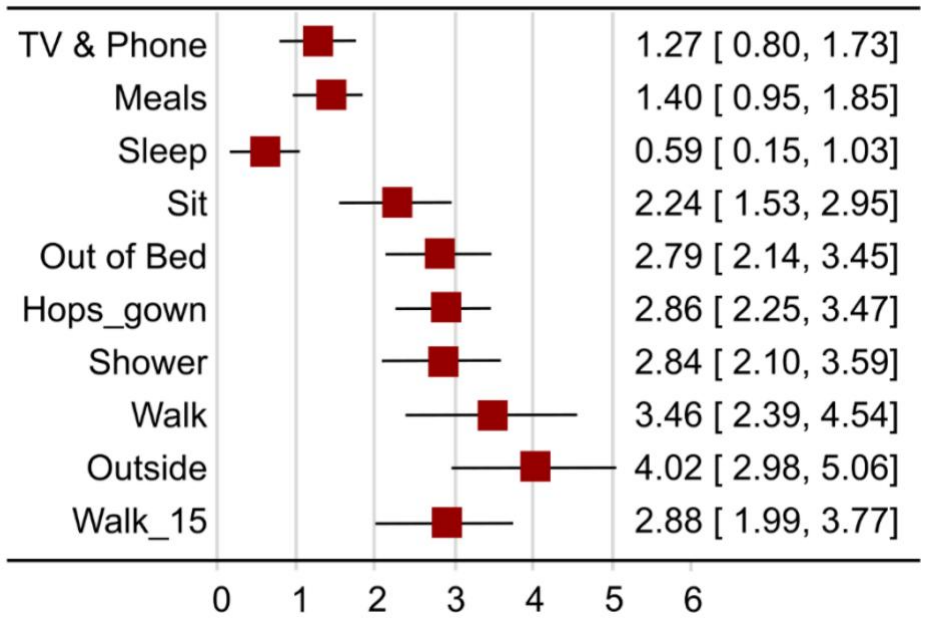
Discrimination parameters for each Functional Status-based Pain Assessment (FSPA) item from the graded-response model (GRM).

Table 4 displays the discrimination for the 4 models, all of which favored the GRM over other models. Figure 2 displays the discrimination power (parameters) of each FSPA item based on the GRM, which we chose. Using GRM, walking outside the room (4.02, 95% CI, 2.98-5.06) exhibited the highest discrimination parameter, followed by walking inside the room (3.46, 95% CI, 2.39-4.54). Conversely, the lowest discrimination values were observed for sleeping (0.59, 95% CI, 0.15-1.03) and watching television, using the phone, or reading (1.27, 95% CI, 0.80-1.73).

**Table 4.** Response value results of 4 model comparisons, using 4 criteria within the item response theory framework.^a^

	AIC	BIC	AICc	CAIC
GRM	11309.02	11518.13	11320.8	11568.13
PCM	11772.08	11943.54	11779.87	11984.54
GPCM	11404.31	11613.42	11416.09	11663.42
RSM	11831.21	11889.76	11832.1	11903.76

a Models with smaller values are preferable to models with larger values.

Abbreviations: AIC = Akaike’s information criterion; AICc = corrected Akaike’s information criterion; BIC = Bayesian information criterion; CAIC = consistent Akaike’s information criterion; GPCM = generalized partial credit model; GRM = Graded response model; PCM = partial credit model; RSM = rating scale model.

**Figure 3.**
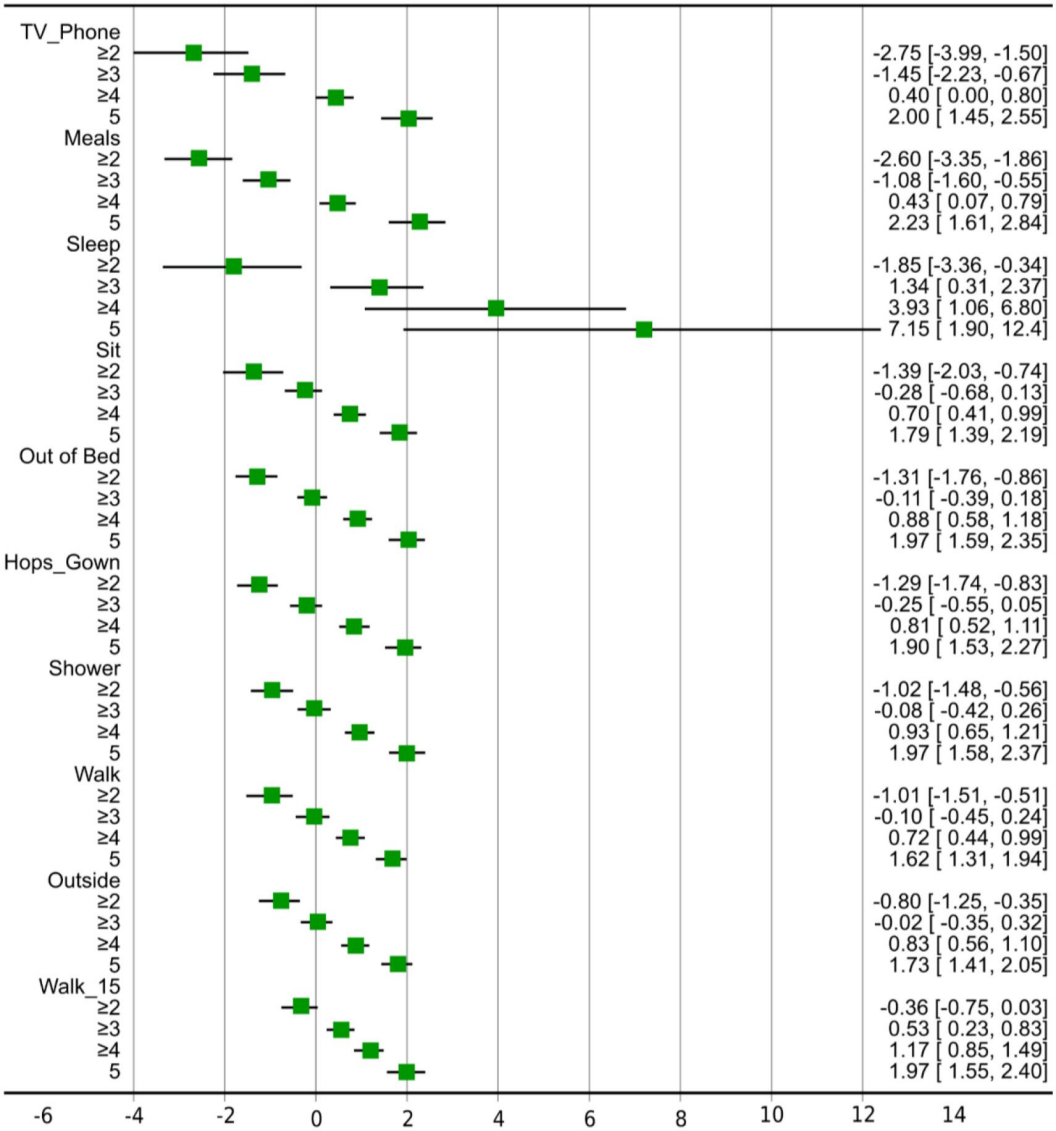
Difficulty parameter for each item from the graded response model (GRM).

**Figure 4.**
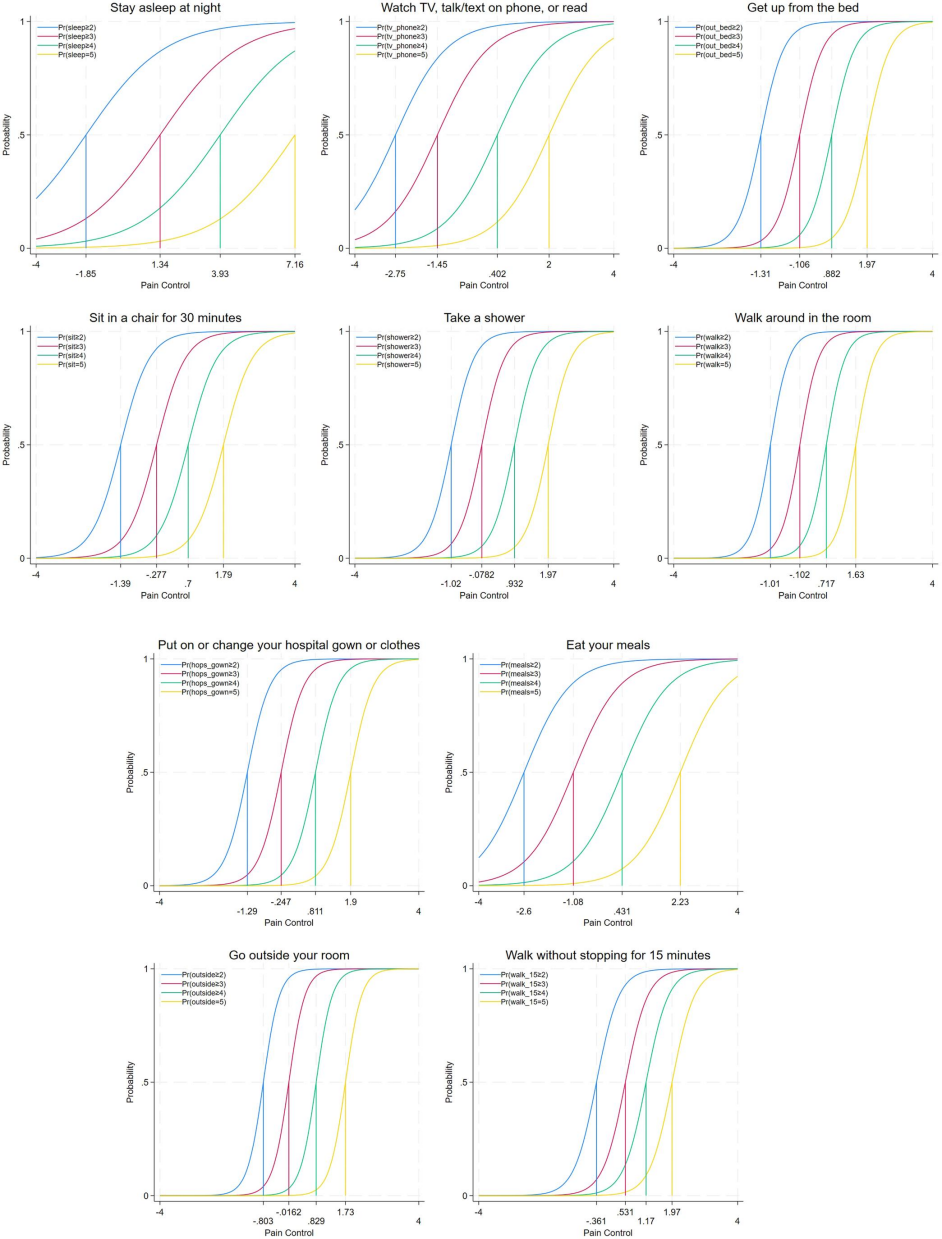
Graphs of the boundary characteristics curve for each item of the Functional Status-based Pain Assessment (FSPA). A steeper slope means higher discrimination while a slow-rising slope means lower discrimination. The value of 0.50 probability on the *y*-axis at which the pain control (latent trait or theta) is crossed for each item gives the difficulty level for that item at that response level. Item “outside” had the highest discrimination value. Probability = the likelihood of item endorsement based on difficulty and pain control level.

Figure 3 shows that, for the difficulty parameter, items exhibited variations both within and across their respective thresholds or response categories. Item difficulty denotes the pain control level at which there is a 50% chance of responding to that or a higher category of activity level. Figure 3 shows that, for example, a pain control level below −2.75 SD was associated with a 50% likelihood of endorsing the response “very difficult.” There was a 50% chance of endorsing the response “difficult” or a higher response for item “Watch TV, talk/text on the phone, or read,” signifying very poor pain control. Among all items, the ability to use the television or phone was achievable at the lowest pain control level, while walking for 15 minutes necessitated the highest pain control for the first response threshold (“very difficult”). Conversely, walking inside the room necessitated the lowest pain control, while sleeping required the highest pain control for the last response threshold (“very easy”). Figure 4 shows boundary characteristic curves for each item of the FSPA. These offer a visual representation of the discrimination and difficulty parameters. Most items, excluding sleep, effectively discriminated within-item responses, as evidenced by the steepness of their slopes.

**Figure 5.**
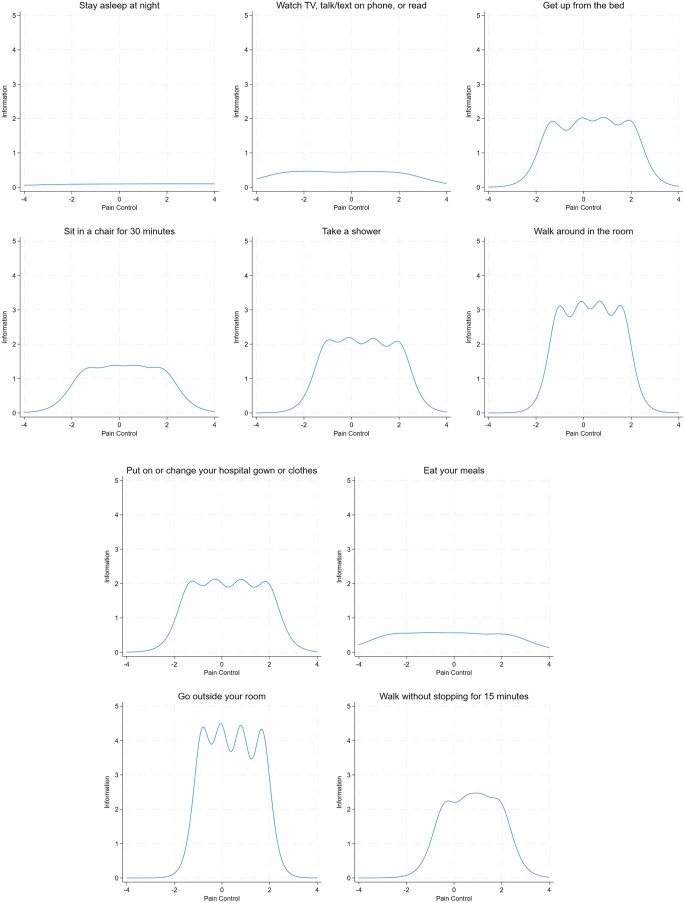
Functional Status-based Pain Assessment (FSPA) item information function curves. Precision of an item across its latent trait (pain control in this case) is presented as curve that is shaped by its difficulty and discrimination.

**Figure 6.**
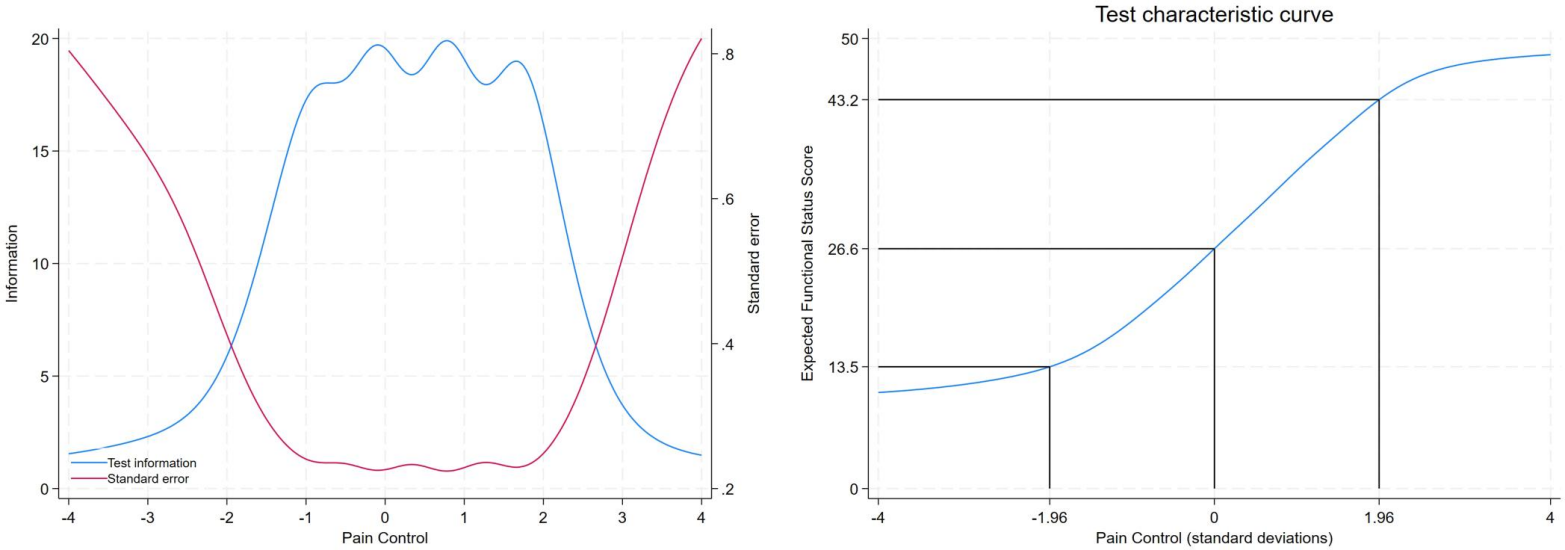
Left panel: Test information curve. Information = the overall precision of the scale presented as a blue curve that is shaped by its difficulty and discrimination across pain control level. Standard error, the inverse of information variance, is represented by a red curve. Right panel: Test characteristic curve: Expected score on the whole test based on the sum of probabilities is plotted against the pain control level.

Figure 5 shows the contribution of items in estimating pain control along the pain control continuum, explored through the item information function (IFF). The IFF offers a measure of an item’s reliability or precision; more reliable items will gauge pain control around the estimated difficulty parameter with greater precision. Figure 5 reveals variations in reliability across items, with some item information concentrating within an area between −2 SD and 2 SD around the mean, while other items exhibit almost a uniform distribution around the mean. For instance, the item “Go outside your room” exhibited the highest amount of information between −2.0 SD and 2.5 SD around the mean, whereas the item “Stay asleep at night” displayed a flatter item information curve. Figure 6, left panel, displays the test information function, which serves as a measure of the FSPAs overall reliability. The Test information function provided the most information when pain control scores ranged between −2 SD and 3 SD around the mean. The test characteristic curve depicted a non-linear sigmoid-shaped relationship between FSPA score and pain control, wherein an increase in pain control correlated with increased scores. Figure 6, right panel, shows that, based on our data, patients with average pain control would score approximately 27 on the FSPA, while 95% of patients would score between 13 and 43.

### Comparisons, modeling of FSPA versus mean pain: on NRS

The correlation between the mean NRS score for all responses and the mean FSPA was −0.4342 (*P* < .0001).

### Cronbachs’ alpha

Reliability of the FSPA showed a Cronbach’s alpha of 0.91, far over the 0.80 limit traditionally considered a strong value. Dropping any particular item of the FSPA did not greatly alter the Cronbach’s alpha, which remained above 0.80 regardless of which item was dropped.

### Factor analysis

**Figure 7.**
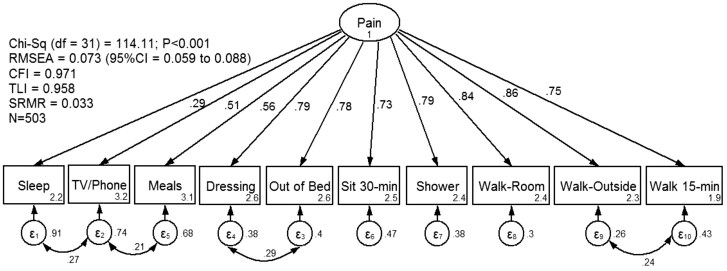
Final confirmatory factor analysis model for the FSPA Questionnaire. Standardized loadings are reported here. CFI = comparative fit index; FSPA = Functional Status-based Pain Assessment; RMSEA = root mean-square error of approximation; SRMR = standardized root-mean squared residual; TLI = Tucker–Lewis index.

Principal component factor analysis found that the first factor explained 56% of the variance, and the scree plot suggested a one-factor solution. Using CFA, we found that all items, except sleep, strongly loaded on this factor with range from 0.60 for use of TV or phone to 0.85 for going outside the room; sleep item factor loading was 0.38. Global fit indices were initially unsatisfactory but improved with adding covariances to the model; overall, these results confirmed a single-factor model (Figure 7).

## DISCUSSION

We developed and demonstrated the feasibility of administering a daily inpatient pain assessment tool based on the daily functional status of adult inpatients with SCD.

We demonstrated strong internal validity of the FSPA. The Cronbach’s alpha remained above 0.8 regardless of which item we dropped from the survey. Correlation between concurrently measured daily NRS pain intensity scores and daily FSPA scores was moderate and highly significant. Overall, the PCFA results were consistent with our conceptual hypothesis that all the items of the FSPA tap into 1 dimension. Further, the CFA eigenvalues for this survey combined would add up to 10. Since 5.57 out of the 10 was explained by the first factor, the first factor explained 57.7% of the variance in the FPSA score. Last, the CFI was 0.93 or significantly better than a null model of no relationship between items. Traditionally, a value above 0.95 is considered adequate for CFI.

Together, these analyses demonstrate good preliminary development and validation of the FSPA survey. While validation is not complete, the FSPA appears to be a brief assessment tool with strong internal validity, explained by a single conceptual factor we think relates to readiness for discharge for a SCD VOC, and related to salient measures such as pain intensity, the traditional objective measure of readiness for discharge.

Our items are more brief than the longer YAFFAQ, and the YAPFAQ is aimed at children, whereas the FSPA is aimed at adults.

A few important limitations of our study deserve note. One limitation of our study is including more than 1 survey from a patient; we incorporated repeated measures into our analysis without statistical adjustment. Surveys from the same patient might induce a higher-than-real correlation. Second, we faced logistical challenges, mentioned in “Procedures, Measures,” which led to mismatch of lab data, hospital identifiers, and survey data for some patients and exclusions of those patients as a result. We were unable to retrieve and repair these mismatched lab-hospital id-survey pairs because of a hospital switch from 1 electronic health record vendor to a second vendor during the study. However, we used laboratory data only for study population characteristics and not as part of the survey analyses. The missingness in lab data does not affect the internal validity of our survey results, but does affect their generalizability.

We note there is work on several multidimensional pain assessments, which measure experiences and dimensions over time.^13-15^ However, the recall for these assessments is usually weeks to months, making them inappropriate for daily assessment. We note a recent review^16^ that described functional scales used in systemic lupus erythematosus, but that the scales with the shortest recall periods still required a week of recall.^17-20^ We note also a study of a fibromyalgia functional scale, which used a recall period of a few days.^21^ Zempsky et al. also developed a pain burden assessment.^22^

Current recommendations for measuring pain dimensionality over time for patients with SCD can be found in the PhenX toolbox and other validated measures.^23-26^ Among these, we did not find a tool focused on inpatient pain variability in SCD adults, with a short, daily recall period.

We therefore maintain the FPSA can become the adult correlate of Zempsky’s YAPFAQ, helpful for assessing readiness for hospital discharge during a VOC, and perhaps even for predicting readiness. We believe the FSPA could be used daily to improve pain communication between adult SCD VOC patients and their inpatient clinicians, could enhance assessment of readiness for discharge from a functional perspective, and perhaps even could predict discharge from the hospital. If validated, the FPSA could also perhaps guide more scientific, rather than empiric, approaches to inpatient pain therapy, approaches that focus on functional impediments to discharge from the hospital due to pain. Future research should determine if FSPA use may potentially decrease length of stay and decrease hospitalization costs, by aiding the judgment and negotiation of readiness for discharge of SCD patients.

## Data Availability

Data used for these analyses are archived at a VCU file storage facility, and may be released, depending on the purpose, upon request of the first (contact) author. Please allow 2-4 weeks for processing time.
